# The Correlation between Shrinkage and Acoustic Emission Signals in Early Age Concrete

**DOI:** 10.3390/ma15155389

**Published:** 2022-08-05

**Authors:** Magdalena Bacharz, Kamil Bacharz, Wiesław Trąmpczyński

**Affiliations:** Department of Concrete Construction and Geotechnics, Faculty of Civil Engineering and Architecture, Kielce University of Technology, 25-314 Kielce, Poland

**Keywords:** new diagnostic method, concrete, early age damage, acoustic emission, shrinkage, NDT

## Abstract

This study analysed the processes of damage formation and development in early age unloaded concrete using the acoustic emission method (IADP). These are of great importance in the context of the durability and reliability of a structure, as they contribute to reducing its failure-free operation time. Concrete made with basalt aggregate and Portland or metallurgical cement cured under different conditions after demoulding was the test material. The obtained damage values were compared with the measured concrete shrinkage, and a shrinkage strain–acoustic emission signal (resulting from damage) correlation was found. The correlation allows easy measurement of damage level in the early period of concrete hardening, and consequently can be the basis of a non-destructive method.

## 1. Introduction

Concrete, especially reinforced concrete (RC), is currently the most commonly used material for the construction of buildings and engineering structures. It is subjected to various effects, i.e., mechanical loads (static and dynamic) [1,2], variable temperature [3,4] and humidity, as well as chemical and biological attacks, contributing to the corrosion of these objects [5,6].

An important aspect is not only the current strength of elements or entire structures but also their durability [7].

Increasingly complex and ambitious design challenges and sustainable development goals require using concrete and steel with new properties that significantly impact the behaviour of structures [8,9]. Tight construction schedules, which is another factor influencing structural durability, have increased the attractiveness of prefabrication. The practice of assembling components in a factory has helped improve the quality and durability of the structures being built. Since prefabricated components require a high degree of manufacturing accuracy, poor supervision may affect the durability of mass-produced elements, as experience shows [10]. Manufacturing errors in newly constructed buildings [11,12] impact their performance [13,14], leading to extensive and costly repairs.

Undoubtedly, the width and growth of cracks are crucial parameters in diagnosing buildings [15]. For this reason, cracks in concrete [16,17] and reinforced concrete [18,19,20] are still extensively studied and analysed. Furthermore, the description of cracks is constantly modified and developed due to the heterogeneity of concrete and complex states of stress and strain that accompany cracking and microcracking [21,22,23].

The cracks observed in the structures are the final result of a more complex process of the formation and development of damage in early age concrete [24]. When hardening, concrete reduces its volume due to moisture loss and chemical processes, referred to as shrinkage. Shrinkage is often discussed in the literature [25] not only as a phenomenon that takes place in early-age concrete [14,25] but also as a factor impacting structural durability [26]. Various calculation models have been developed to determine shrinkage strain as another type of load acting on a structure [27,28,29,30]. Shrinkage is responsible for cracking that may lead to fatigue, shortened service life, or compromised load capacity. Extensive research is performed to mitigate early microcracking in concrete by adding fly ash or slag cement and using lightweight aggregate, as described in [31,32,33]. Another approach to mitigate microcracks is to incorporate nanomaterials. For example, carbon nanotubes were found to reduce the microcracks and shrinkage [34].

Stress concentration, caused by heterogeneous and often excessive temperature and humidity fields, is also a factor in microcracking [14,35]. Microcracks can transform into cracks observed on the surface of structures [36], thereby contributing to the ingress of water and other aggressive substances that have an adverse effect on durability. A possible solution to this issue may be the use of carbon nanotubes, although it requires additional analysis regarding structural concretes [37].

Non-destructive or semi-destructive methods [38,39,40,41] are essential in diagnosing concrete. Chemical (qualitative and quantitative) analyses, physical (optical, thermographic, radiological, acoustic, electromagnetic) methods, or biological methods (macroscopy, microscopy) allow the assessment of strength and homogeneity (sclerometric and acoustic methods), location of defects and damage (acoustic and radiological methods), location of reinforcement, corrosion assessment (electromagnetic, radiological, electrical methods), and the evaluation of humidity and temperature distribution (indirect—physical, chemical).

One of the non-destructive methods (used in this paper) is the acoustic emission (AE) method—IADP (identification of active destructive processes), which has been successfully used to analyse the development of cracks resulting from loads on the structure, including the service load [42,43,44,45,46,47]. AE methods are applied in analysing the parameters of elastic (acoustic) waves generated in the material during the cracking process [48,49,50,51].

The objective of this paper is to analyse AE signals caused by destructive processes in unloaded early age concrete, detected using the AE IADP technique, and their correlation with the measured shrinkage strains.

In the papers [52,53], AE signals were assigned to the destructive processes identified and tracked using the IADP technique. The authors of [54,55] verified the suitability of IADP for testing destructive processes in unloaded concrete hardening at various temperatures and under different maintenance conditions, taking into account the type of cement and aggregate and the presence of reinforcement and admixtures.

This paper attempts to determine the correlation between the strain values and the destructive processes recorded in the hardening concrete. The tests were performed on unloaded concrete that varied in the type of cement and maintenance conditions after removal from the moulds. The strains and the course of the destructive processes in concrete were tested for 56 days. Furthermore, the shrinkage strains were estimated according to three standard approaches: Eurocode 2 (EC2) [27], Model Code 90–99 (MC90–99) [28], and Bazant Baweja—model B3 (B3) [29]. The influence of maintenance conditions on the level of predicted strains was analysed. A robust correlation was observed and described between the number of destructive processes, their energy, and the shrinkage strains. This means that by knowing (measuring) early age concrete shrinkage strains, one can determine the AE signal range as the basis for determining whether in addition to the basic processes and other processes indicative of progressive internal micro-damage occurring in concrete, thereby reducing structural durability and reliability. The tests and their analyses are a continuation of the work aimed at developing a universal non-destructive method for assessing early age concrete.

## 2. Materials and Methods

**Figure 1:**
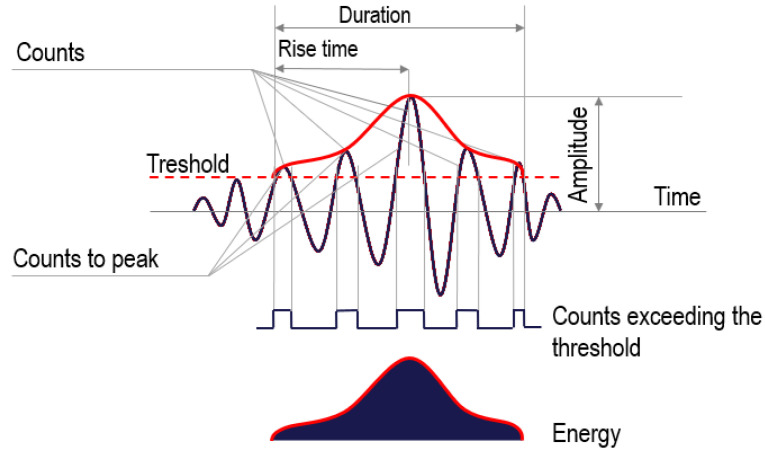
Acoustic signal parameters.

Destructive processes in the material (microcracks, crack propagation, and dislocations) are accompanied by a rapid release of energy, generating elastic (acoustic) waves in the material. These waves (Figure 1) gradually disappear as a result of energy absorption in the thermal process.

In this study, the IADP method was used to measure destructive processes. The method does not rely on the selected parameters of the AE signal, but is based on the reference signals created with the use of 12 parameters of the recorded electric signal: counts, counts to peak, duration, rise time, amplitude in mV or dB, energy, strength, root mean square, mean level, mean frequency, reverberation frequency, and initial frequency. The use of this method for the evaluation of reinforced concrete structures (bridges) is presented in [45,46,47].

The signal is measured when an active destructive process occurs in a given element during the measurement, e.g., a crack is formed or propagated [56]. Damage in the element but not developing does not generate AE signals. Acoustic waves generated in the material can be recorded using acoustic sensors (usually piezoelectric). Their proper arrangement enables the localisation of their source. The selected signal parameters are also analysed [57] and can be used to determine the type of failure [49,50,51].

Using AE signals, it is also possible to analyse other destruction processes. In the case of prestressed elements, these are [42,43]: microcracking, friction between crack faces, initiation and growth of cracks, cracking at the concrete reinforcement interface, concrete spalling, friction at the concrete reinforcement interface, corrosion, plastic deformation, and cracking of cables and other reinforcement. This method was successfully used in diagnosing prestressed concrete elements and structures [44].

In [53,55], the IADP method was used to analyse the failure processes in the early phase of concrete hardening. Destructive (12 signal parameters) were assigned to following classes of reference signals [47,52]:

- microcracks in the cement paste and at the aggregate-paste interface (Class 1),
- internal propagation of microcracks (Class 2),
- formation of microcracks on the concrete surface (Class 3), and
- growth of microcracks (Class 4).

A destructive process in concrete (for example, microcracks in the cement paste) is an acoustic wave sources. Twelve parameters of the AE wave (signal) are recorded by the AE sensors and compared with the base of reference signals, which allows us to determine the destructive process (in this case, Class 1) and its location based on the arrival time of the AE signal.

Although microcracks and damage in early age concrete do not have a direct impact on the safety of the structure [21] (unless their size exceeds a certain level [26,58]), they affect the durability of the structure because they become the sites of future crack initiation.

### 2.1. Test Elements

Nine concrete samples (three series of three samples) with dimensions of 150 × 150 × 600 mm were used in the tests. The samples varied in terms of selected parameters described below and summarised in Table 1. The samples were made with basalt aggregate 2–16 mm from the Gracze quarry and different cement types. In sample C−I (MC) CEM I 42.5N MSR/NA, Portland cement from the WARTA cement plant was used, while in samples C−III (MC) and C−III (AC) CEM III/A 42.5N—LH/HSR/NA, metallurgical cement from the Górażdże cement plant was used. After removal from the moulds, C−III (MC) samples were cured at 100% humidity for 10 days and then subjected to proper tests, i.e., measurements of strains and volume change, and AE signal recording. The C−III (AC) samples were tested without prior curing in water. The moisture-cured samples (C−III (MC) and C−I (MC)) and the non-moist-cured sample (C−III (AC)) hardened at a constant temperature of 22 ± 2 °C for 56 days.

The composition of individual concrete mixes is shown in Table 2.

### 2.2. Research Methods

#### 2.2.1. Strain Measurements and Prediction

**Figure 2:**
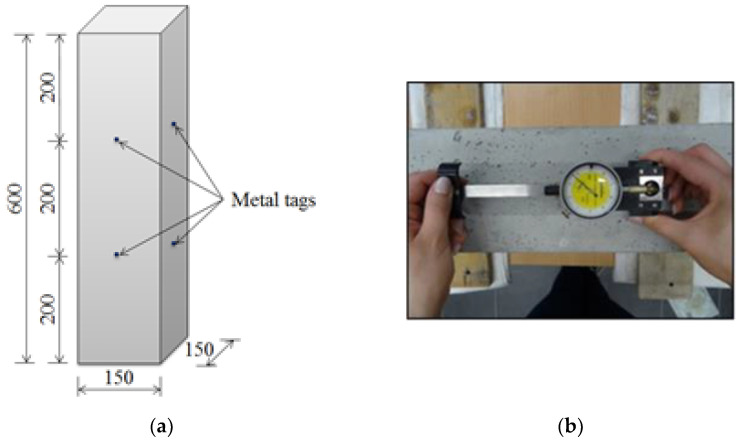
(**a**) Spacing of metal tags for strain measurements, (**b**) measurement of strain with an extensometer.

An 8 inch (~20 cm) demountable mechanical strain gauge was used for the strain test (Figure 2b). The measurement points were steel elements glued to the four walls of the sample, as shown in Figure 2a.

**Figure 3:**
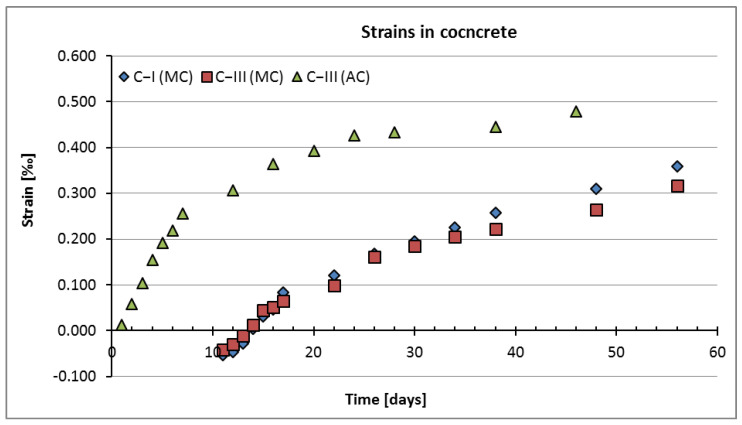
The result of the strain test of specimens from series C−I (MC), C−III (MC), and C−III (AC).

Strains were measured on four faces of each specimen and the basic statistical parameters were calculated, i.e., mean value, coefficient of variation, and variance. The strain results obtained for three series of specimens at varied curing conditions are shown in Figure 3, where the vertical axis shows the results of shrinkage strains and the horizontal axis shows the time, in days, for which the measurements were made. It also should be noted that during the test, the samples were not additionally loaded.

**Figure 4:**
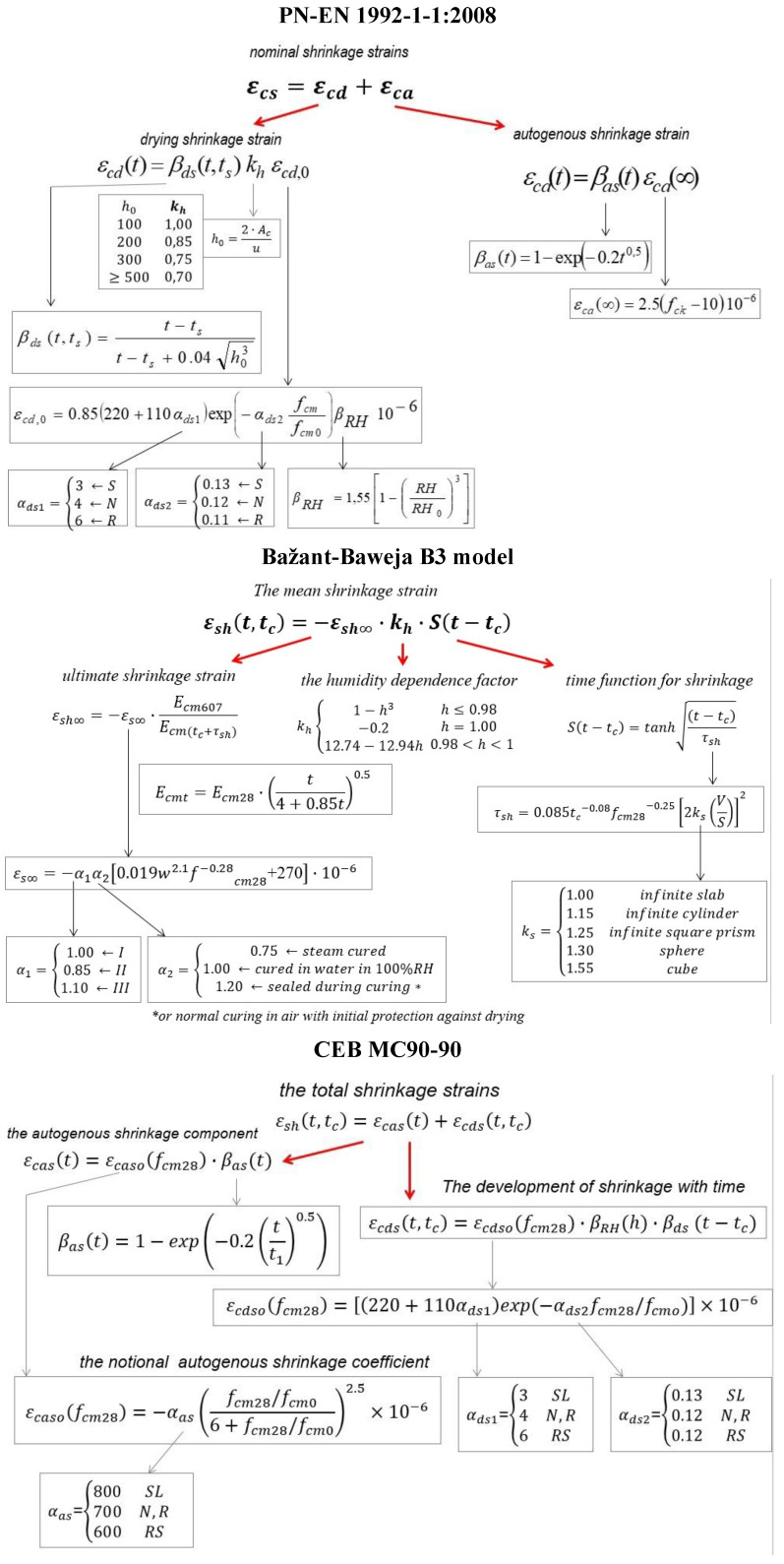
Procedures for predicting shrinkage strain according to selected standards.

The strains (shrinkage) from the tests were compared with the results predicted according to the following standards: Eurocode 2 (EC2)—PN-EN−1992−1−1 [27], Model Code 90–99 (MC) [28], and the Bazant-Baweja approach (B3) [29]. The adopted assumptions are given in Figure 4.

**Figure 5:**
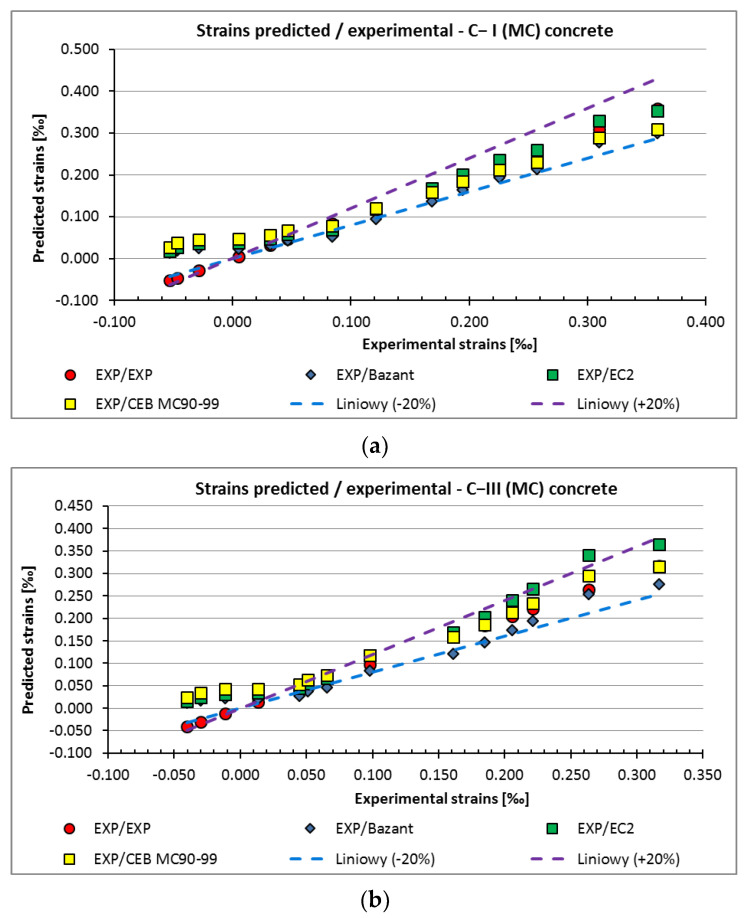
The strains in the pre-cured samples: (**a**) C−I (MC), (**b**) C−III (MC).

**Figure 6:**
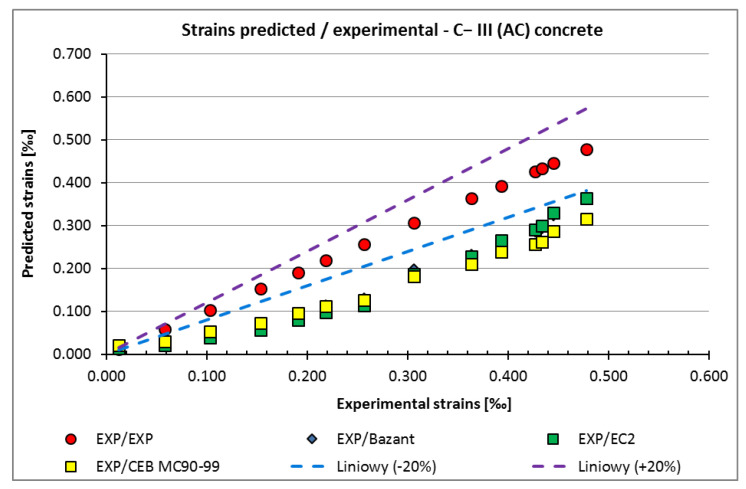
The strain results of non-cured C−III (AC) samples.

The strain values obtained in the C−I (MC) and C−III (MC) concrete (subjected to 10-day curing after removal from the moulds) are shown in Figure 5a,b, and for C−IIII (AC) (no curing after demoulding) are shown in Figure 6.

The strains estimated according to the EC2, MC90–99, and B3 approaches in water-cured samples C−I (MC) (with Portland cement) and C−III (MC) (with metallurgical cement) fell within the range of +/− 20% in relation to the values obtained in the laboratory tests. In the first week of the test, swelling of concrete C−I (MC) and C−III (MC) was observed. The phenomenon was most likely due to the higher humidity in the thermal chamber than the ambient humidity in the laboratory hall where the samples were prepared for testing.

Higher ambient humidity in the climatic chamber resulted from samples with higher humidity being placed there. With the same number of samples and the volume of the climatic chamber, the result was a significant increase in humidity inside the chamber. As a result, the humidity of the samples themselves increased, and, as a result, their volume increased. Over time, the humidity decreased, contributing to the recorded shrinkage deformations, not swelling. This was largely due to the low humidity of the environment in which the chamber itself was located, which contributed to the final decrease in the level of humidity.

In the case of the non-moisture-cured C−III (AC) concrete with metallurgical cement, the strain values predicted according to the standards were lower than the values obtained from laboratory tests.

The impact of not curing and curing errors in the initial stage of hardening on the shrinkage strains are not taken into account in the adopted standards [27,28,29], which may be the reason for the differences (Figure 6).

#### 2.2.2. Measurements of Destructive Processes—IADP Method

**Figure 7:**
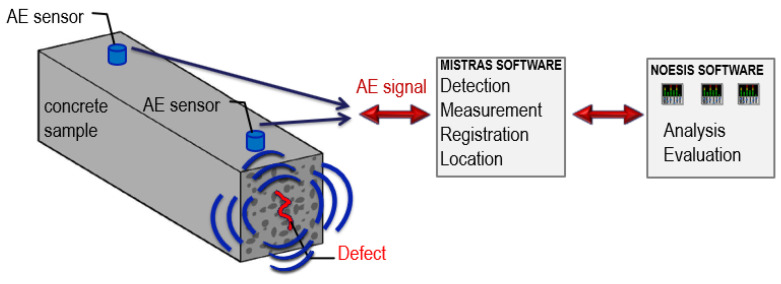
The concept of the IADP method for early-age concrete.

The tests were carried out using the IADP acoustic emission method, validated, among others, in [53]. The flow chart of the method is shown in Figure 7.

Concrete samples C−I (MC) and C−III (MC) were removed from the moulds after 10 days of cure. C−III (AC) samples were tested without curing. Two Vallen VS30-V sensors were placed on one side of each sample. The concrete surface was cleaned before the sensors were attached to the surface with a thermally conductive silicone paste and elastic rubber.

**Figure 8:**
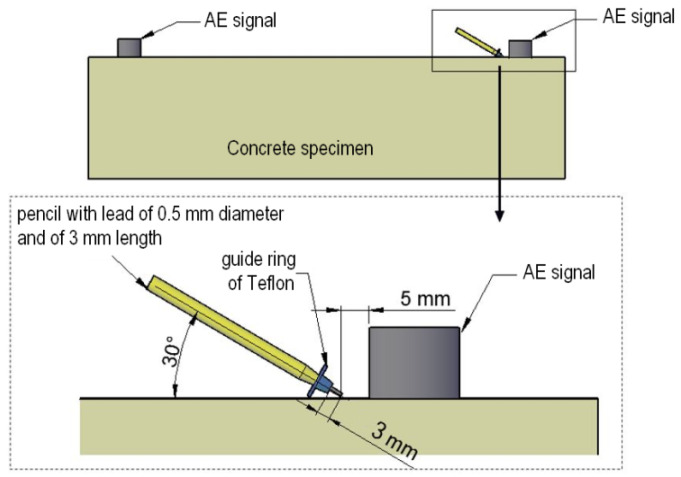
Sensor calibration before testing—Hsu Nielsen source.

The calibration before the start of the test consisted of checking the amplitude of the recorded AE signal from breaking the graphite of a pencil with a hardness of 2H, diameter 0.3 mm, length 3 mm (Hsu Nielsen source [59,60]) inclined to the surface of the sample at an angle of 30° (Figure 8).

If the amplitude of the signal excited by breaking the pencil recorded on each sensor reached 100 dB, calibration was performed successfully. If a lower amplitude was recorded, the amount of thermal paste was added to improve the contact between the sensor and the concrete surface.

Before the test, an input data file was created in the Mistras program. The dimensions of the tested element, the spacing, and number of AE sensors as well as measurement parameters were set. AE signals were recorded in the samples in 12 h cycles preceding the measurement at 1–8, 12, 16, 20, 24, 28, 38, and 46 days at the points where strain values were taken, the samples were weighed, and cracks were observed.

The test results were analysed using Noesis software and the reference signal database [53]. Based on that, the recorded signals were assigned to three classes that defined individual processes using the supervised approach.

The classification involved comparing the registered AE signals with the previously prepared reference base. It was developed in the research described in [54].They were based on the division of a broad database into 3 groups of signals (classes) on the basis of the analysis of 12 parameters describing the AE signal. The exact process of creating databases is presented in [43,49,54].

## 3. Results

### 3.1. Number of AE Signals Analysis

**Figure 9:**
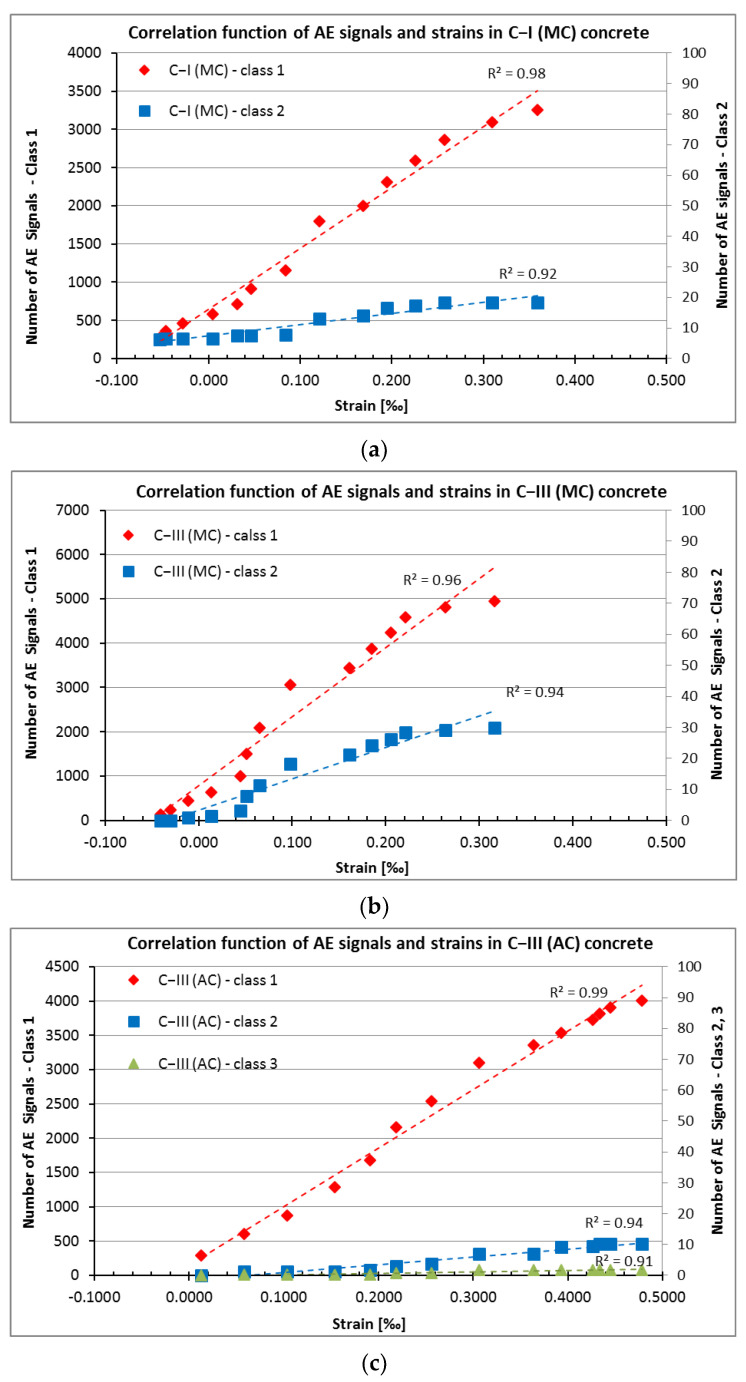
Correlation functions of AE signals and strains for the C−I (MC) (**a**) and C−III (MC) concrete series (subjected to curing) (**b**) and C−III (AC) (without curing) (**c**).

The correlation between the strains obtained from the tests and the number of AE signals (destructive processes such as microcracks in the cement paste and at the aggregate-paste interface (Class 1), the internal microcrack propagation (Class 2), and the formation of microcracks on the concrete surface (Class 3) was recorded in concrete series C−I (MC)–C−III (AC), and as shown in Figure 9a–c.

In the presented graphs on the horizontal axis, the values of shrinkage strains are shown, while the vertical axes show the number of class 1 signals on the left axis, and the number of class 2 and 3 signals on the right axis. The use of two vertical axes is due to significant differences between the quantities of class 1, 2, and 3 signals. Presenting them on one axis would make their analysis impossible. However, if class 3 signals were not recorded in a sample, no additional vertical axis was added.

From the results in Figure 9, it follows that there is a very strong correlation, practically linear, between the strain values and the number of destructive processes recorded in the sample: microcracks in the cement paste and at the grain boundaries (Class 1), the development of microcracks in the cement paste (Class 2), and the formation of microcracks on the concrete surface (Class 3).

Equations of linear functions shown in Figure 9 are summarised in Table 3.

### 3.2. AE Signal Energy Analysis

In a paper [53], the unit energy values of the signals of individual classes (1, 2, and 3) were determined for concrete series C−III (MC) and C−III (AC). These values, summarised in Table 4, were used to determine an increase in the signal energy in C−I (MC)-C−III (AC).

**Figure 10:**
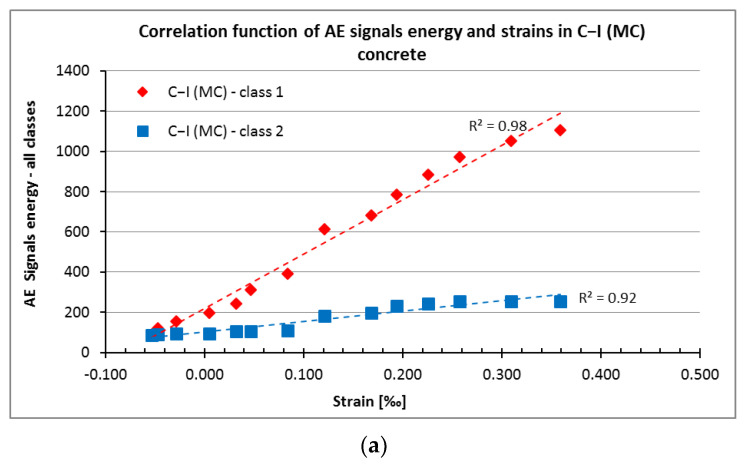

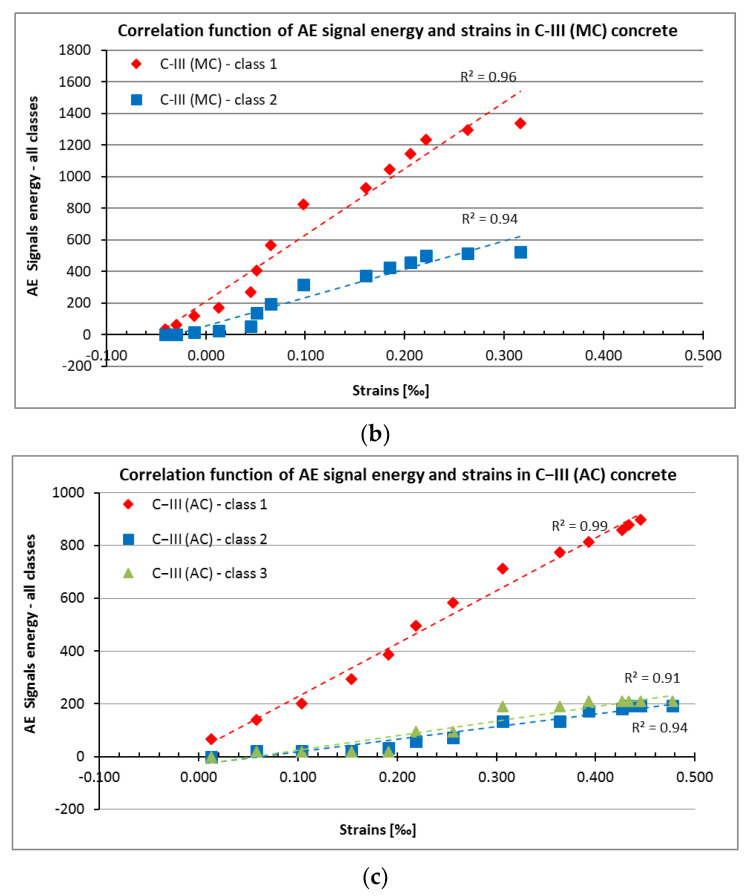
Correlation functions of AE signals energy and strains in samples subjected to curing (**a**) C−I (MC), (**b**) C−III (MC), and (**c**) C−III (AC) (without prior curing).

The obtained correlation between the unit energy of the signals of individual classes (representing damage) and the strains is shown in Figure 10.

From the results in Figure 9, it follows that there is a very strong linear correlation between the increase in strains and energy of destructive processes recorded for the samples.

The equations of linear functions shown in Figure 10 are summarised in Table 5.

### 3.3. Correlation Function of Strains and Destructive Processes in Unloaded Concrete

One of the objectives of the study was an attempt to develop a relationship that would allow the prediction of the number of AE signals due to damage and processes in time.

On the basis of the trend lines in the graphs showing the increase in both shrinkage strains and acoustic emission signals, a natural logarithm function was adopted as the base function modified with adjustment coefficients.

(1) $$y=\alpha \cdot A_{SK}\cdot \ln\left(x\right)+\beta \cdot B_{SK}$$

where:

*α*, *β*—estimated correction factors,

*A_sk_*, *B_sk_*—coefficients of the logarithmic trend line of the additional shrinkage strain for the tested sample, and

*x*—time in days.

Correction factors, *α* and *β*, were adopted to better match the results. The coefficients were estimated as the ratios of the *A* and *B* coefficients read from the trend line (logarithm functions) of the increase in shrinkage strains (*A_SK_* and *B_SK_*) and the acoustic emission signals (*A_AE_* and *B_AE_*).

(2) $$\alpha =\frac{A_{AE}}{A_{SK}},\beta =\frac{B_{AE}}{B_{SK}}$$

The values of the *α* and *β* coefficients were estimated with two methods (API and APII) based on the results obtained from the concrete series. In the first approach (API), the *A_AE_/A_SK_* and *B_AE_/B_SK_* ratios were determined for each concrete series (C−I (MC) to C−III (AC) separately, and then the values were averaged, obtaining *α* and *β* coefficients, respectively. The second approach (APII) consisted of determining the average *A_AE_*, *A_SK_*, *B_AE_*, and B_SK_ coefficients from the C−I (MC) to C−III (AC) concrete series. Then, *α* and *β* coefficients were calculated from *A_AE_/A_SK_* and *B_AE_/B_SK_* relationships. The parameters adopted to estimate the theoretical values of AE signal increase are presented in Table 6.

**Figure 11:**
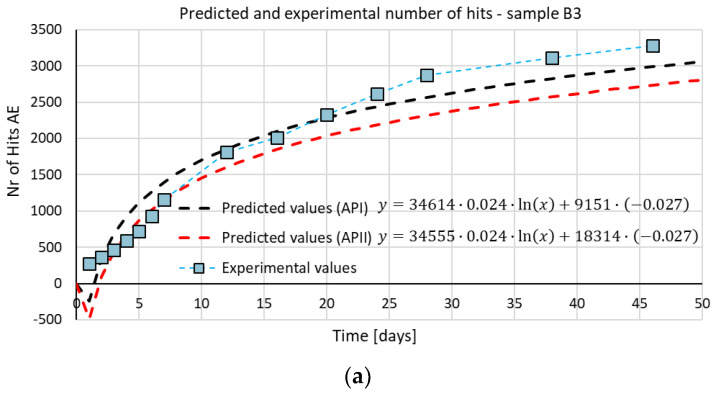

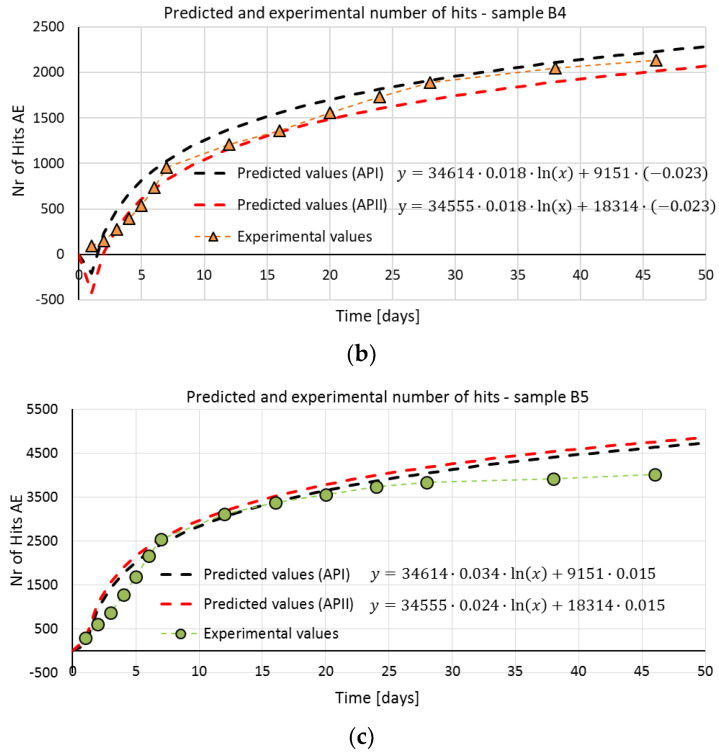
The results of the number of hits predicted (based on strain measurements) and recorded in samples: (**a**) C−I (MC), (**b**) C−III (MC) (series subjected to curing) and (**c**) C−III (AC).

The initial verification of the results was carried out based on the calculated coefficients (α and β) and the values of experimental shrinkage strains from the C−I (MC) to C−III (AC) concrete series. The course of the predicted increase in acoustic emission signals over time in relation to their experimental values is shown in Figure 11. The graphs show the increase in the number of AE signals for all three classes of AE signals.

The dotted lines (with markers at measurement points) show the real increase in AE signals for the C−I (MC) to C−III (AC) concrete series over time, while the dashed lines show the increase in predicted course of the AE signals. The black dashed line represents the theoretical gain estimated from the α_I_ and β_I_ coefficients, while the dashed red line represents the theoretical gain of the AE signals estimated from the α_II_ and β_II_ correction factors. Both lines were developed on the basis of the shrinkage strains of the examined concrete, i.e., the trend line in the diagram of the actual strain increment. The analysis showed that the predicted values of the increase in the number of AE signals were consistent with those obtained experimentally and, therefore, the adopted algorithm of the procedure can be considered properly matched.

As has been shown, it is possible to describe the number of AE signals as a function of shrinkage strains, which is the basis for the wide testing program taking into account various parameters that affect the shrinkage in concrete.

## 4. Discussion

Using the AE technique, a very strong correlation, in the range of 0.91 to 0.99, between the strain values and the number of AE signals related to destructive processes in early age concrete was found. Additionally, the recorded processes were divided into three classes, i.e., microcracks in the cement paste and at the grain boundaries in the cement paste (Class 1), the formation of microcracks in the cement paste (Class 2), and the formation of microcracks on the concrete surface (Class 3). The indicated correlation was described using a simple logarithm function.

Additionally, a very strong correlation was found between the increase in strain and the increase in energy of the AE signals, which can be described using a similar logarithm function.

Hence, by measuring shrinkage strains and knowing experimentally defined factors, one can estimate the theoretical range of AE signals. On this basis, one can determine whether, in addition to the basic processes, there are also those indicative of progressive internal micro-damage. This is important, as they can reduce the structure’s durability and reliability.

Additionally, a comparative analysis of experimental shrinkage strains and strains estimated according to selected calculation models, including those used in the relative standards shown in Figure 5 and Figure 6, was made. The values estimated according to approaches EC2, MC 90–99, and B3 are within the +/− 20% range of these obtained in laboratory tests for concrete subjected to 10-day water curing after demoulding (C−I (MC)) with Portland cement and with metallurgical cement (C−III (MC)). However, in the case of concrete with metallurgical cement hardening at the declared temperature without prior curing in water (C−III (AC)), the strain values predicted according to the standards are lower than those obtained from the laboratory tests. This may be due to the fact that no guidelines are available in the adopted standards for estimating the impact of curing errors or lack of curing in the initial period of concrete hardening on the values of shrinkage strains.

## 5. Conclusions

Presented results show that:

- The increase in the number of AE signals resulting from damage:
  o Class 1—microcracks in the cement paste and at the aggregate-paste interface,
  o Class 2—internal propagation of microcracks, and
  o Class 3—formation of microcracks on the concrete surface

is strongly correlated with the increase in strain over time and can be described by logarithmic function. Simple logarithm functions describing this relation were developed for C−I (MC), C−III (MC), and C−III (AC) concrete with specific parameters and variables.

- The increase in the energy of Class 1, 2, and 3 signals over time is strongly correlated with the increase in strain over time and functions describing this relation were developed.
- The results presented may form the basis for simple diagnostics of new elements. It means that by knowing the early age shrinkage strains (easy to measure), one can estimate the early age damage.
- More concrete tests should be performed to optimise the function formula and other variables should be added, e.g., aggregate, admixtures, and concrete strength.
- It was also shown that:
- Experimental shrinkage strains and those estimated according to selected standards show a strong correlation in the case of cured concrete C−I (MC) and C−III (MC)).
- In the case of non-cured concrete (C−III (AC)), the shrinkage strains estimated according to the standards are lower than those measured in the laboratory tests.

The obtained results are very promising, in particular due to the diagnostic possibilities they offer. For this reason, an extended program to verify these findings on other specimens made with a different concrete composition is being prepared.

## Figures and Tables

**Table 1:** Characteristics of concrete samples.

Symbol	Aggregate	Cement	Hardening Conditions	Temperature Condition
C−I (MC)	Basalt	CEM I	10 days of wet curing	Constant
C−III (MC)	Basalt	CEM III	10 days of wet curing	Constant
C−III (AC)	Basalt	CEM III	drying in air	Constant

**Table 2:** Composition of concrete mixtures [kg/m^3^].

Symbol	Basalt 2–8	Basalt 8–16	Sand 0–2	CEM I	CEM III	Water
C−I (MC)	581	731	691	360	x	180
C−III (MC)	581	731	691	x	360	180
C−III (AC)	581	731	691	x	360	180

**Table 3:** Equations of linear functions of the correlation between strains and number of hits.

Concrete Series	Class 1	Class 2	Class 3
C−I (MC)	y = 8000x + 650	y = 35x + 7	
C−III (MC)	y = 4200x + 210	y = 1800x + 55	
C−III (AC)	y = 8800x + 110	y = 25x − 1.5	y = 5x − 0.5

**Table 4:** The unit energy values.

Unit Energy	C−I (MC)	C−III (MC)	C−III (AC)
Class 1	0.34	0.27	0.23
Class 2	14.05	17.57	18.99
Class 3			113.65

**Table 5:** Equations of linear functions of the correlation between strains and energy of AE signals.

Concrete Series	Class 1	Class 2	Class 3
C−I (MC)	y = 2700x + 220	y = 515x + 100	
C−III (MC)	y = 15,600x + 785	y = 100x + 3	
C−III (AC)	y = 2000x + 25	y = 480x − 30	y = 555x − 30

**Table 6:** Parameters determined with two methods.

Concrete Series	*A_SK_*	*B_SK_*	*A_EA_*	*B_EA_*	API		APII	
					α_I_	β_I_	α_II_	β_II_
C−I (MC)	0.024	−0.027	909	−388	34.614	9151	34.555	18.314
C−III (MC)	0.018	−0.023	612	−270				
C−III (AC)	0.034	0.015	1129	18				

## Data Availability

The data presented in this study are available in: M. Bacharz, Wykorzystanie metody emisji akustycznej do badania procesów destrukcyjnych w betonie nieobciążonym, Ph.D. Thesis, Politechnika Swietorzyska, Kielce, Poland, 2016. (In Polish).
